# Effects of NAA and BAP, Double-Layered Media, and Light Distance on *In Vitro* Regeneration of *Nelumbo nucifera* Gaertn. (Lotus), an Aquatic Edible Plant

**DOI:** 10.1155/2014/745148

**Published:** 2014-05-07

**Authors:** Noraini Mahmad, Rosna Mat Taha, Rashidi Othman, Azani Saleh, Nor Azlina Hasbullah, Hashimah Elias

**Affiliations:** ^1^Institute of Biological Sciences, Faculty of Science, University of Malaya, 50603 Kuala Lumpur, Malaysia; ^2^International Institute for Halal Research and Training (INHART), Herbarium Unit, Department of Landscape Architecture (KAED), International Islamic University Malaysia, 53100 Kuala Lumpur, Malaysia; ^3^Faculty of Applied Science, MARA University of Technology, 40450 Shah Alam, Selangor, Malaysia; ^4^Department of Agriculture Sciences, Faculty of Technical and Vocational Education, Sultan Idris Education University, 35900 Tanjung Malim, Perak, Malaysia

## Abstract

*In vitro* direct regeneration of *Nelumbo nucifera* Gaertn. was successfully achieved from immature explants (yellow plumule) cultured on a solid MS media supplemented with combinations of 0.5 mg/L BAP and 1.5 mg/L NAA which resulted in 16.00 ± 0.30 number of shoots per explant and exhibited a new characteristic of layered multiple shoots, while normal roots formed on the solid MS basal media. The double-layered media gave the highest number of shoots per explant with a ratio of 2 : 1 (liquid to solid) with a mean number of 16.67 ± 0.23 shoots per explant with the formation of primary and secondary roots from immature explants. In the study involving light distance, the tallest shoot (16.67 ± 0.23 mm) obtained from the immature explants was at a light distance of 200 mm from the source of inflorescent light (1000 lux). The plantlets were successfully acclimatized in clay loam soil after 8 months being maintained under *in vitro* conditions.

## 1. Introduction

Lotus is in the genus of* Nelumbo* and belongs to the family of Nelumbonaceae. The Nelumbonaceae family consists of a perennial aquatic and emergent angiosperm plant which consists of two species:* Nelumbo nucifera* Gaertn. (the Asian or sacred lotus) and* Nelumbo lutea* (Willd.) Pers. (the American lotus or water chinquapin). The former is distributed in Asia and North Australia and the latter is found in North and South America [1, 2]. Lotus is an important economic aquatic plant, not only as a dainty and ornamental flower but also as a source of herbal medicine, with strong bioactive ingredients, including alkaloids and flavonoids, and antioxidant, antisteroidal, antipyretic, anticancerous, antiviral, and antiobesity properties [3–6]. Lotus is usually propagated vegetatively through rhizome division or tuber production, but the normal propagation rate is very low [7]. It can also be multiplied through seeds but, for quick and more efficient germination, the seeds need to be scarified by rubbing the outer hard seed coat gently on sandpaper at both ends and finally immersing them in water to initiate germination. Scarified seeds were germinated after 3-4 days while normal seeds took 10–15 days to germinate. If the hard coating remains intact, the seeds will remain viable for centuries and it may take a few years for the seed to sprout if placed in water [8].

The present research is aimed at studying* in vitro* regeneration of immature (yellow plumule) explants on solid Murashige and Skoog (MS) media supplemented with different combinations and concentrations of *α*-napthaleneacetic acid (NAA) and 6-benzyl aminopurine (BAP). To date, this is the first report of successful* in vitro* regeneration from immature explants (yellow plumule) with new characteristics (layered multishoots) in double-layered MS media. At the same time, the study focused on the effects of light distances on the* in vitro* regeneration of this species.

## 2. Materials and Methods

### 2.1. Plant Material

*Nelumbo nucifera* Gaertn. plants were obtained from a natural lake, the Chini Lake, in Pahang, Malaysia. No specific permits were required for the described field studies. The location is not privately owned or protected in any way and the field studies did not involve endangered or protected species.

### 2.2. Seed Sterilisation and Germination

Immature seeds were collected after two months of anthesis, from intact plants. The immature seeds (0.5–1.0 cm) with bright yellow colour were obtained from enclosed flowers. The healthy seeds were oval-shaped and initially washed with tap water and teepol. The seeds were then sterilised with 99% (v/v) sodium hypochlorite solution for 1 min and rinsed with distilled water three times. In a laminar flow cabinet, the seeds were dipped in 70% (v/v) ethanol for 1 minute and blotted with sterile tissue. The cotyledons were excised into two and the yellow plumules were cut (3 mm) and cultured with adaxial polarity on solid basal MS (Duchefa) medium [9] supplemented with 30 g/L sucrose (System) and 8 g/L agar (Oxoid).

### 2.3. *In Vitro* Plant Regeneration and Culture Conditions

Growth regulators, *α*-napthaleneacetic acid (NAA), and 6-benzyl aminopurine (BAP) were dissolved in sodium hydroxide (NaOH) and added to Murashige and Skoog (MS) media. After two weeks, the plumules were excised into small pieces (3 mm^2^) and cultured on MS media with 30 different combinations and concentrations of NAA (Sigma) and BAP (Sigma). Thirty replicates for each treatment were used. The pH was adjusted to 5.5 by adding 0.1 M of either sodium hydroxide (NaOH) or hydrochloric acid (HCl). Finally, the media were set to pH 5.5 and autoclaved at 104 kPa (15 Psi^2^) at 121°C for 21 minutes. The sterilised media were poured into 1/3 of 60 mL sterile containers. All cultures were incubated in a culture room at 25 ± 1°C, with a 16-hour photoperiod with 1.496 W m^−2^ of light intensity. Subcultures were performed every 21–28 days to provide new and fresh nutrients under the same conditions.

### 2.4. Effects of Double-Layered MS Medium

The completely regenerated plantlets (6–8 weeks) with shoots and roots were transferred onto solid MS media supplemented with a hormone media (bottom layer) and liquid MS basal media (upper layer). Solid media were fixed to 1 cm height in the sterile tubes. At the same time the liquid media level was altered to a ratio of either 1 : 1, 1 : 2, or 1 : 3 to a solid level.

### 2.5. Effects of Light Distance

Cultures were also exposed to the light source at different distances (55 cm, 10 cm, 15 cm, 20 cm, 25 cm, and 30 cm). Cultures were incubated at 25 ± 1°C with 16 hours of light and 8 hours of dark. Thirty replicates for each treatment were prepared.

### 2.6. Acclimatization

Eight-month-old* in vitro* plantlets which were cultured on MS medium supplemented with 1.5 mg/L BAP and 0.5 mg/L NAA were gently washed under running tap water. For the first 2-3 weeks, plantlets were transferred to clay loam soil and covered with plastics in the culture room. Subsequently, plantlets were exposed to the natural environment in the green house. In this experiment, observations on lotus growth in fresh water aquarium (clear medium) and 50 cm diameters of clay containers were done. The development of lotus parts (roots, petioles, stems, and leaves) was examined after seven-day interval.

## 3. Statistical Analysis

All the experiments were repeated trice and thirty replicates were used. The effect of different treatments was quantified as mean ± SE and the data were subjected to statistical analysis using Duncan's multiple range test (DMRT) at 5% level of significance [10].

## 4. Results and Discussion

**Figure 1:**
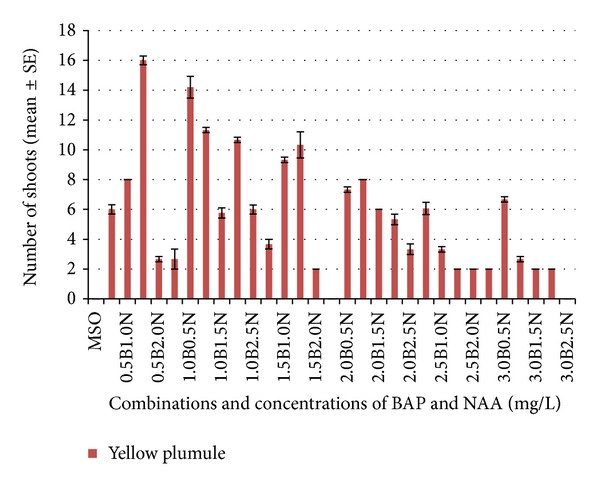
Effects of combinations and concentrations of BAP and NAA on lotus shoots formation from yellow plumule explants.

**Figure 2:**
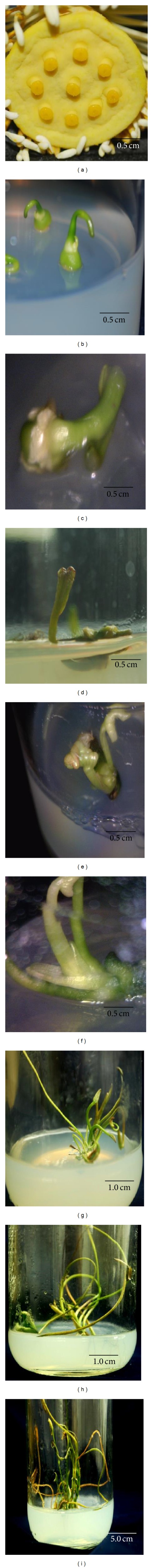
Effects of BAP and NAA on shoot formation from the immature explants (yellow plumule) of* N. nucifera* after 24 weeks in culture on MS medium. Yellow plumule cultured on MS solid media supplemented with 0.5 mg/L BAP and 1.5 mg/L NAA. The cultures were maintained at 25 ± 1°C with 16 hours of light and 8 hours of dark, with 1000 lux intensity of light for 24 weeks. (a) Yellow pod with nine immature seeds. (b) Two hinged shoots (explants) on. (c) First subculture after 2 weeks cultured on solid MS basal (control). (d) Elongation of first shoot. (e) Two rolled shoots after 3 weeks. (f) Formation of layered shoots after 4 weeks. (g) Elongation of shoots after 6 weeks. (h) Formation of first unrolled leaf after 8 weeks. (i) Layered multiple shoots (vertical position) with formation of primary roots after 12 weeks.

Figures 1 and 2 show the responses from immature (yellow) plumule explants cultured in 30 different combinations and concentrations of BAP and NAA on solid MS media.  Figure 1 also shows that the highest number of shoots per explant was found when yellow explants were cultured on MS media supplemented with combinations of 0.5 mg/L BAP and 1.5 mg/L NAA, resulting in 16.00 ± 0.30 shoots per explant. In contrast, no root formation was observed in all treatments from yellow explants. The root formation occurred 2–4 weeks after transferring to solid MS media. No abnormal shoot formation was seen in all the treatments of yellow explants. Shou et al. [7] also reported that shoots derived from lotus buds which were cultured on MS medium containing 0.5–2.0 *μ*M NAA, 0.2% activated charcoal, with or without 0.1 *μ*M BA for 1 week, had to be transferred onto MS basal medium for 4 weeks for root induction and resulted in 9.2 ± 0.7 number of roots per explant.

The regeneration of yellow plumules resulted in a new shoots characteristic, with smaller and lighter green vertically layered shoot and root formation (only primary). Overall, the combination range of 0.5–1.0 mg/L BAP with 0.5–2.5 mg/L NAA was optimum for immature explants and gave the highest (2–16) number of shoots per explant. Among the treatments, the same concentration ratios of BAP and NAA (0.5 mg/L BAP and 0.5 mg/L NAA, 1.0 mg/L BAP and 1.0 mg/L NAA, 1.5 mg/L BAP and 1.5 mg/L NAA, 2.0 mg/L BAP and 2.0 mg/L NAA, and 2.5 mg/L BAP and 2.5 mg/L NAA) were recognised to respond vigorously for yellow plumules explants (immature). Subcultures were needed every 21–28 days to maintain the freshness of the media and for the accumulation of shoots and roots. Shou et al. [7] reported that the maximum number of shoots was induced from lotus bud explants on MS medium containing 8 g/L agar, 30 g/L sucrose, and 4.44 *μ*M benzyladenine (BA) added with 0.54 *μ*M *α*-naphthalene acetic acid (NAA) for 4 weeks, with low rates of lotus multiplication (3.50 ± 0.05 number of shoots per bud). The different results in the current study show a shorter period of response (2–4 weeks), which were influenced by the types of explants and ratios between NAA and BAP and cytokinin and auxin and were considered critical factors for* in vitro *shoot multiplication [11, 12].

Media that were rich in nutrients such as Murashige and Skoog [9] were shown to promote vitrification in some plant species [13, 14], and root formation occurred with the addition of auxin at lower concentrations, while a higher concentration of cytokinin was found to induce the formation of shoots.

The present study showed that the explants (yellow plumules) cultured on the optimum solid MS media supplemented with 0.5 mg/L BAP and 1.5 mg/L NAA for 20 weeks developed a new shoots characteristic (layered multiple shoots). However, no abnormal shoot formation was seen in the treatment of explants. Possibly, a new variety or some somaclonal variants had occurred when the double-layered media were used. The effect of this double-layered media could be beneficial for producing new varieties of lotus in the future. Considering* Nelumbo nucifera* Gaertn. as an aquatic plant, double-layered media are necessary to make it possible to grow this plant as a terrestrial plant for ornamental, medicinal, therapeutic, and aromatic purposes. In fact, by introducing this type of media (double-layered media) mass propagation of this species can be achievable. Up to our knowledge, this is the first report of maintaining* Nelumbo nucifera* Gaertn.* in vitro* for at least 5–8 months in double-layered media, whereas in complete liquid or solid media,* in vitro *regeneration could not be maintained more than 3-4 weeks. The capability to retain* in vitro* growth is useful and beneficial for nurseries with deduction of 5-6 times of maintenance.

**Figure 3:**
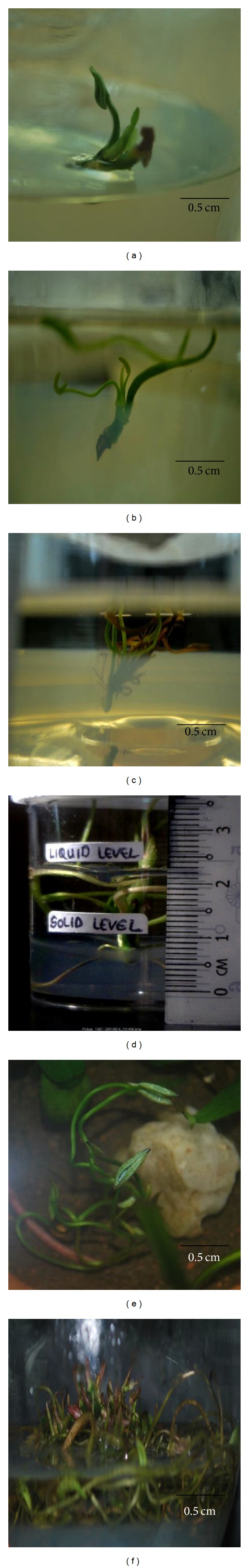
Effects of yellow plumule of* N. nucifera* on double-layered media (on solid MS media and liquid MS media supplemented with 0.5 mg/L BAP and 1.5 mg/L NAA). (a) Formation of the first and second shoots. (b) Elongation of the first three shoots. (c) Layered multiple shoots after 6 weeks. (d) Formation of roots after 4 weeks of subculture. (e) Elongation of leaves after 7 weeks. (f) Layered multiple shoots after 10 weeks.

Table 1 and Figure 3 show the effects of solid and liquid levels in the regeneration of explants from yellow seeds. As a control, explants were cultured on solid MS basal media. Yellow shoots elongated and turned to green colour, with a mean number of 9.10 ± 0.51 shoots per explant and formed primary roots. The highest number of shoots per explant was found in the liquid to solid ratio of 2 : 1 with a mean of 16.67 ± 0.23 shoots per explant and the formation of primary and secondary roots for explants from yellow seeds (Figure 3(c)), with the formation of layered multiple shoots. In media containing a liquid to solid ratio of 1 : 1, yellow shoots elongated normally with a mean of 15.67 ± 0.09 shoots per explant (Figure 3(d)). Even though the lotus is an aquatic plant, in a liquid to solid ratio of 3 : 1 (flooded), shoots turned brown with a mean of 10.33 ± 0.23 shoots per explant for yellow shoots. The better contact between explants and the liquid medium increased the availability of cytokinin and the ability for nutrient uptake [15], increased the dilution of any exudates from explants in the liquid medium [16], and made the aeration in the liquid medium more efficient and sufficient, which enhanced both growth and multiplication of shoots [17].

**Figure 4:**
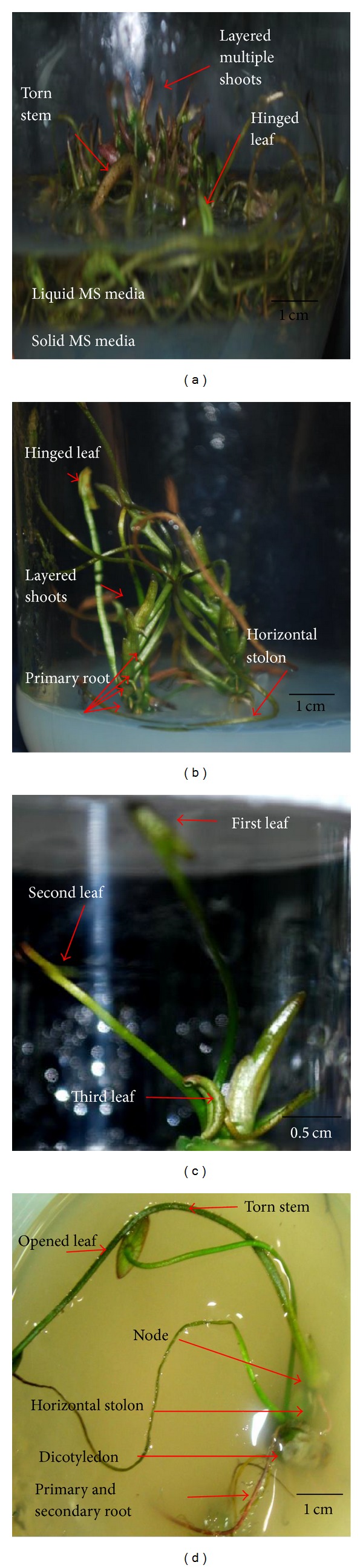
Comparison of plantlets morphology of* N. nucifera* from various conditions of growth. (a)* In vitro* multiple shoots with new shoots characteristic from immature explants (yellow plumule) on double-layered MS media. (b) Layered multiple shoots transferred to solid MS media for root formation. (c) Normal shoots elongated on MS media for root formation. (d)* In vivo* grown lotus after 4 months.

Table 2 and Figure 4 illustrate the effects of the distance of the light source on the multiplication of lotus shoots. The tallest or the highest height of shoots (16.67 ± 0.23 mm) obtained from yellow plumule explants was at a distance of 200 mm, and the shortest or the lowest height of shoots (9.12 ± 0.51 mm) was at 50 mm. All of the explants in the nearest light distance (0.0 mm) were dried out and died. From this experiment, a light distance of 100–200 mm was found to be optimum for the healthiest growth of shoots and plantlets from yellow plumule explants.

**Figure 5:**
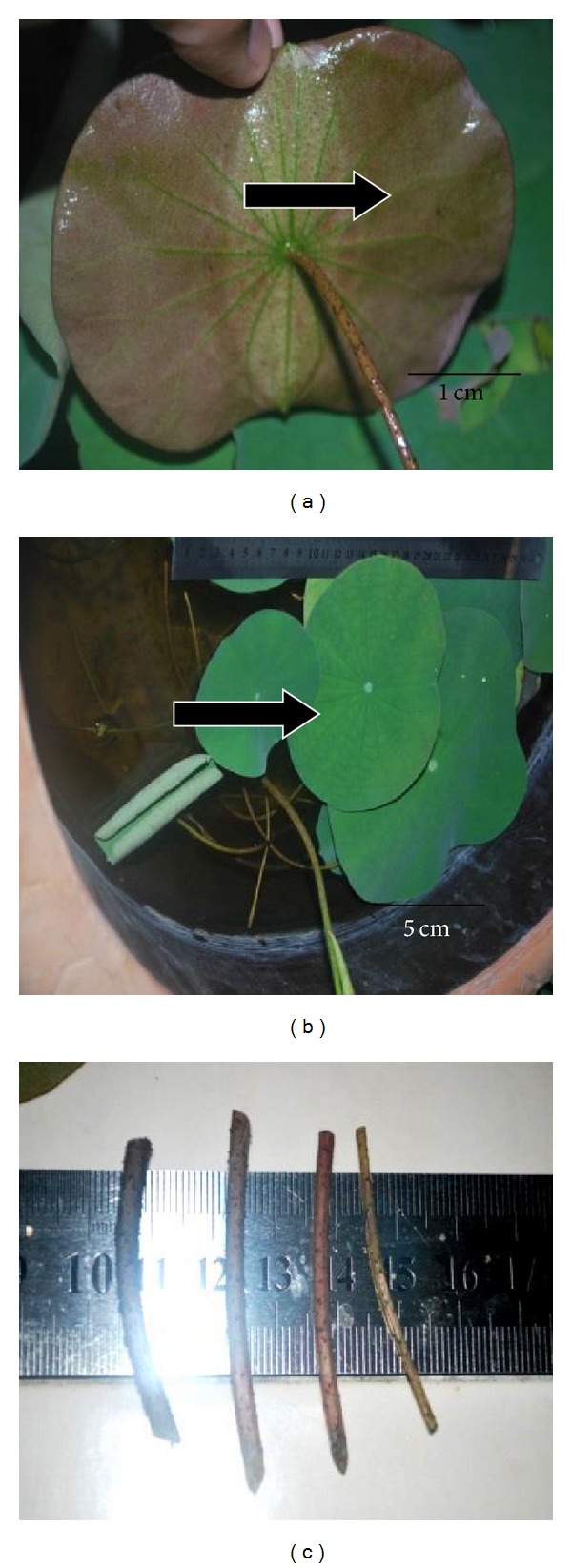
First 4 leaves of 8-month-old acclimatized plantlets emerged from water surface. (a) Adaxial of leaves: red spotted. (b) Abaxial of leaves: waxy green. (c) Stem: hollow and prickles (tiny torns).

**Figure 6:**
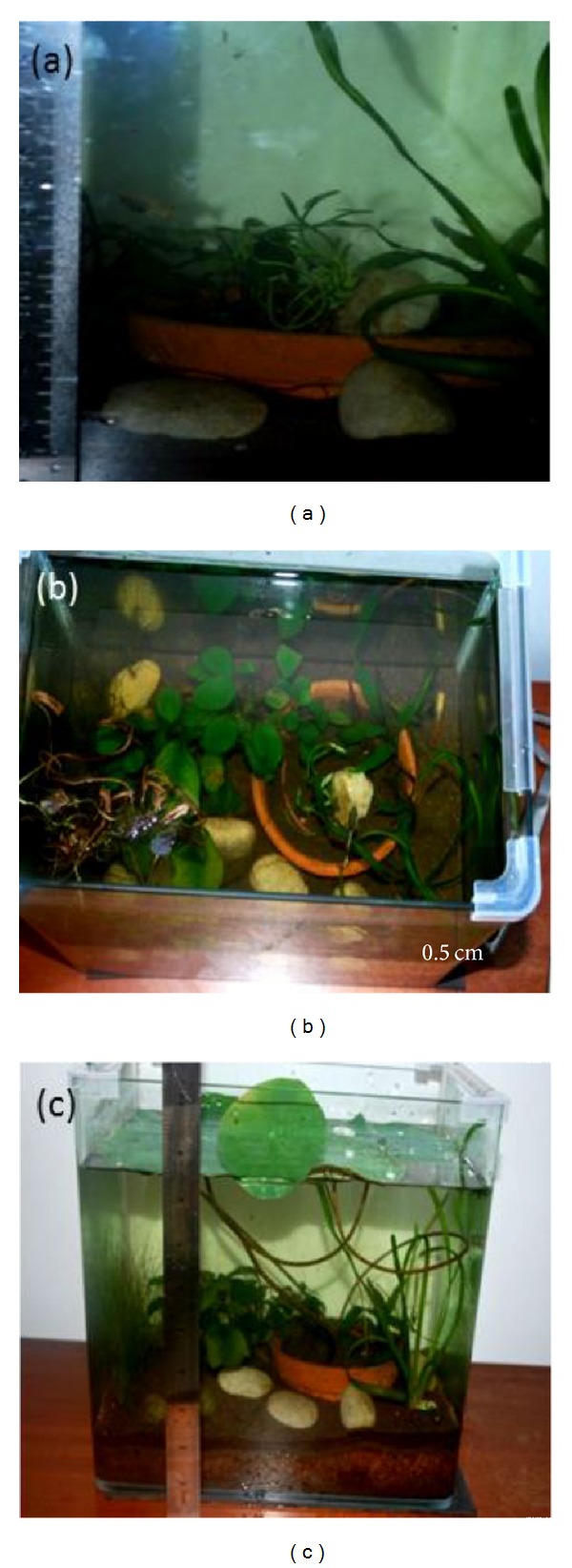
Acclimatization in fresh water aquarium mixed with black clay loam soil and associated with guppy fish. (a) Eight-month-old plantlets. (b) Shoots elongation. (c) Plantlets after 8 months of acclimatization.

**Figure 7:**
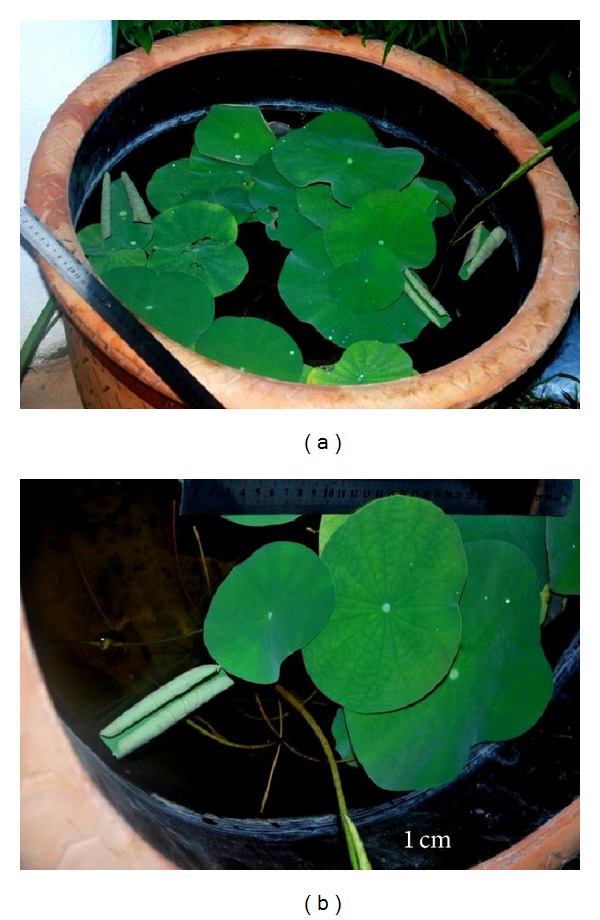
Eight-month-old plantlets being acclimatized in containers. (a) Green floating leaves. (b) First 3 leaves (emerged), 1 rolled leaf, and 1 petiole.

Table 3 and Figure 5 show the acclimatization development of plantlets transferred to fresh water aquarium (Figure 6) and water container. For the first 8 months being acclimatized, stems and leaves grew vigorously with red spotted leaves and hollow stems. However, when the leaves emerged from water surface, the leaves became fully green in colour. In Figure 7, due to the increasing leaf sizes, the plants were required to be transferred to a larger container (1.0 m^2^).

## 5. Conclusion

Up to our knowledge, this is the first report indicating the successful culture of immature explants (yellow plumules) on optimum double-layered media, consisting of solid MS supplemented with 0.5 mg/L BAP and 1.5 mg/L NAA for 20 weeks, which developed a new shoot characteristic (layered multiple shoots) at an optimum light distance of 200 mm from the inflorescent light source (1000 lux) which gave the tallest shoot (16.67 ± 0.23 mm) and the highest number of shoots per explant (16.67 ± 0.23).

## Figures and Tables

**Table 1:** The effect of double-layered media on the regeneration of shoots. Cultures were maintained at 25 ± 1°C with 16 hours of light and 8 hours of dark, with 1000 lux intensity of light for 4 weeks.

Ratio liquid : solid (mm)	Number of shoots per explants (Mean ± SE)	Observations
0 : 1 (control)	9.10 ± 0.51	Yellow shoots turned green and elongated. Primary root formation
1 : 1	15.67 ± 0.09	Shoots elongated
2 : 1	16.67 ± 0.23	Layered shoots. Primary root formation
3 : 1	10.33 ± 0.23	Shoots turned brown

*Double-layered medium (yellow shoot): combination of solid MS + 0.5 mg/L BAP + 1.5 mg/L NAA and liquid MS basal.

Each value represents the mean of thirty replicates. Mean ± standard error (SE), replicates (*n* = 30).

**Table 2:** The effect of light distance on the regeneration of shoots. Cultures were maintained at 25 ± 1°C with 16 hours of light and 8 hours of dark, with 1000 lux intensity of light for 4 weeks.

Light distance (mm)	Height of shoot, mm (mean ± SE)	Observations
0.0 (control)	0	Yellow shoots became dried and turned brown
50	9.12 ± 0.51	Yellow shoots turned green
100	10.67 ± 0.09	Yellow shoots turned green
150	13.10 ± 1.01	Yellow shoots turned to green
200	16.67 ± 0.23	Yellow shoots elongated
250	9.13 ± 0.50	Yellow shoots elongated
300	6.33 ± 0.18	Yellow shoots turned brown

Each value represents the mean of thirty replicates. Mean ± standard error (SE), replicates (*n* = 30).

**Table 3:** Growth of stems and leaves of different ages.

Age (month)	Number of stem-leaf (mean ± SE)	Leaves' width (cm) (mean ± SE)	Stem (cm) (mean ± SE)	
			Height	Width
2	4.01 ± 0.31	4.12 ± 0.35	30.32 ± 0.65	0.30 ± 0.01
4	8.52 ± 0.11	8.41 ± 0.67	60.23 ± 0.57	0.61 ± 0.20
6	12.09 ± 0.23	12.50 ± 1.23	90.14 ± 1.71	0.92 ± 0.07
8	16.30 ± 0.34	16.27 ± 0.87	120.65 ± 0.62	1.23 ± 0.81
10	20.11 ± 0.13	20.73 ± 0.36	140.41 ± 0.38	1.31 ± 0.92
12	24.42 ± 0.17	30.12 ± 0.08	150.17 ± 0.04	1.52 ± 0.77

Each value represents the mean of thirty replicates. Mean ± standard error (SE), replicates (*n* = 30).
